# Performance comparison of two reduced-representation based genome-wide marker-discovery strategies in a multi-taxon phylogeographic framework

**DOI:** 10.1038/s41598-020-79778-x

**Published:** 2021-02-17

**Authors:** Philipp Kirschner, Wolfgang Arthofer, Stefanie Pfeifenberger, Eliška Záveská, Peter Schönswetter, The STEPPE Consortium, Božo Frajman, Alexander Gamisch, Andreas Hilpold, Ovidiu Paun, Isabel Sanmartín, Emiliano Trucchi, Florian M. Steiner, Birgit C. Schlick-Steiner

**Affiliations:** 1 Department of Ecology, University of Innsbruck, Technikerstraße 25, 6020 Innsbruck, Austria; 2 Department of Botany, University of Innsbruck, Sternwartestraße 15, 6020 Innsbruck, Austria; 3 Department of Biosciences, University of Salzburg, Hellbrunnerstrasse 34, 5020 Salzburg, Austria; 4 Institute for Alpine Environment, Eurac Research, Drususallee 1/Viale Druso 1, 39100 Bozen/Bolzano, Italy; 5 Department of Botany and Biodiversity Research, University of Vienna, Rennweg 14, 1030 Vienna, Austria; 6 Real Jardín Botánico CSIC, Plaza de Murillo 2, 28014 Madrid, Spain; 7 Department of Life and Environmental Sciences, Marche Polytechnic University, Via Brecce Bianche, 60131 Ancona, Italy

**Keywords:** Evolutionary ecology, Molecular ecology, Conservation biology, Phylogenetics, Genotyping and haplotyping, High-throughput screening

## Abstract

Multi-locus genetic data are pivotal in phylogenetics. Today, high-throughput sequencing (HTS) allows scientists to generate an unprecedented amount of such data from any organism. However, HTS is resource intense and may not be accessible to wide parts of the scientific community. In phylogeography, the use of HTS has concentrated on a few taxonomic groups, and the amount of data used to resolve a phylogeographic pattern often seems arbitrary. We explore the performance of two genetic marker sampling strategies and the effect of marker quantity in a comparative phylogeographic framework focusing on six species (arthropods and plants). The same analyses were applied to data inferred from amplified fragment length polymorphism fingerprinting (AFLP), a cheap, non-HTS based technique that is able to straightforwardly produce several hundred markers, and from restriction site associated DNA sequencing (RADseq), a more expensive, HTS-based technique that produces thousands of single nucleotide polymorphisms. We show that in four of six study species, AFLP leads to results comparable with those of RADseq. While we do not aim to contest the advantages of HTS techniques, we also show that AFLP is a robust technique to delimit evolutionary entities in both plants and animals. The demonstrated similarity of results from the two techniques also strengthens biological conclusions that were based on AFLP data in the past, an important finding given the wide utilization of AFLP over the last decades. We emphasize that whenever the delimitation of evolutionary entities is the central goal, as it is in many fields of biodiversity research, AFLP is still an adequate technique.

## Introduction

Phylogeography has led to large advancements in understanding the spatio-temporal evolution of species and the underlying climatic, geological, and ecological processes^1^. Easy access to molecular-genetic data has propelled large-scale and cross-species phylogeographic studies and offered new insights on long-standing questions such as the postglacial colonization of Europe^2^. As such, phylogeography has become an integral part of biogeographic research in general^3^. Owing to the large effort necessary to obtain multiple, informative genetic markers from the genomes of non-model organisms, many studies had to rely on single or a few genetic markers in the past. As broadly discussed, studies utilizing a single or few genetic markers are affected by incongruences due to, for example, pseudogene amplification^4^, or simply different gene genealogies supporting alternative species trees^5^. As a consequence, many journals important to the field decided to no longer accept such studies (e.g. Molecular Ecology). Today, the phylogenetic resolution at different time scales and on different taxonomic levels that can be achieved via high-throughput sequencing (HTS) data is unprecedented^6^. The need to look for alternative ways to generate multi-locus datasets has been constantever since the dawn of molecular systematics.

**Figure 1:**
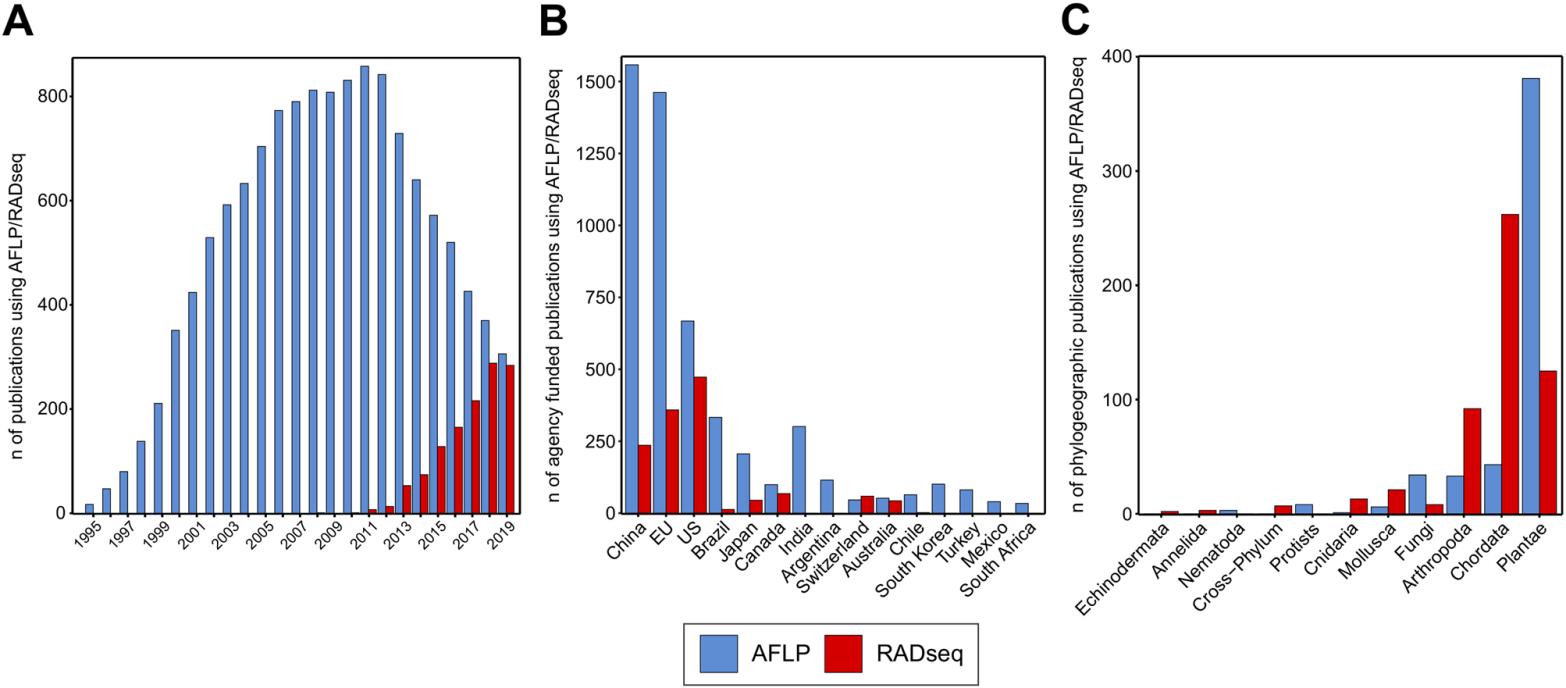
Results of a literature survey on usage, funding, and taxonomic scope of amplified fragment length polymorphism (AFLP, blue bars) and restriction site associated DNA sequencing (RADseq, red bars) studies (details in Supplementary Methods, Supplementary Results). (**A**) Number of publications produced per year using AFLP and RADseq. Total number of studies published until the end of 2019: AFLP, 13,173; RADseq, 1230; introduction of the respective technique (AFLP: 1995; RADseq: 2008) (**B**) Number of publications using AFLP and RADseq grouped by nationality of the funding agency. (**C**) Number of phylogeographic publications using AFLP and RADseq grouped by taxonomic study-group.

An established and widely used method to generate genetic markers from any organism’s genome is amplified fragment length polymorphism (AFLP) fingerprinting^7^. This tool from the pre-HTS era is able to sample several hundred to a few thousand genetic markers from an organism without any prior knowledge of its genome. The method has been extensively used in the field of evolutionary ecology, spanning disciplines from phylogenetics to phylogeography and species delimitation^8,9^. AFLP has been employed for over 20 years, and its applicability has been proven in thousands of studies (Fig. 1A), including also recent publications in highly visible journals^10–13^.

The advancement of molecular methods in the era of HTS helped phylogeography, once data-limited, to become a data-rich field^14^. One HTS-based method, which has been particularly used for phylogeographic studies, is restriction site associated DNA sequencing (RADseq)^15^. This method is able to produce, depending on the experimental design, thousands to tens of thousands of gene fragments that can be used to infer large numbers of single nucleotide polymorphisms (SNPs) from populations of any non-model organism’s genome^16^. The generation of real sequence data that can be analyzed using established models of molecular evolution is a pivotal advantage of RADseq, compared with other marker types such as microsatellites and the above-mentioned AFLP fingerprints. Also, the method’s power in terms of resolution has been empirically demonstrated compared with genotyping methods such as microsatellites or multiple single-locus markers^5,17–20^.

Nevertheless, RADseq is akin to AFLP in terms of discovering markers throughout a genome by subsampling regions targeted by specific restriction enzymes^15^. Methodological limitations and features of both techniques have been thoroughly discussed, and parallels become evident, considering, for example, the problems both methods were reported to have in sampling markers from large, complex genomes^21,22^. Still, RADseq has clear advantages in terms of reproducibility and marker discovery rate^23^, and, as co-dominant sequence data instead of presence-absence data are generated, also a much wider range of applications^16,24^. The mere number of loci available, however, is not always important to answer a biological question. For example, it has been demonstrated that dozens of simple sequence repeats (SSRs) lead to the same result as hundreds to thousands of SNPs^17,20,25^. Such comparisons need to be interpreted carefully, especially if this is done in a phylogenetic context: while models for molecular evolution have been adapted and empirically tested for RADseq derived SNP data^26^, such models are under discussion for SSRs^27^, or not available in case of AFLP. While RADseq is definitely able to resolve fine-scale phylogeographic patterns via large numbers of SNPs^5,28^, the question of how many markers are really needed to do so, has not been answered so far.

A disadvantage of HTS techniques is their resource intensity. While the costs per sequenced base pair are constantly decreasing, a cost shift towards laboratory equipment and computational facilities has occurred that, in some setups, even counterbalances the cost-advantage of decreasing sequencing prices^29,30^. The overall costs depend on the HTS protocol used. Single-digest RADseq protocols, for example, rely on DNA shearing via focused ultra-sonication^15^, which calls for expensive sonicators that are not standard in most labs. Beside other options, one of the most frequently applied and reliable reduced-representation HTS techniques is double-digest RADseq (ddRADseq). In this case, DNA shearing is done enzymatically, which improves the tunability of fragment size selection. To ensure an accurate fragment-size selection, special electrophoresis devices are commonly used for ddRADseq^31^. However, such devices are generally much cheaper than sonicators, and ddRADseq has been emphasized to be more cost-efficient than single-digest RADseq^31^. Generally, large purchases can be avoided by outsourcing steps that depend on expensive machinery to the sequencing companies for additional service charges.

Resource imbalances around the globe, and therefore imbalances in the access to cutting-edge technologies like HTS do matter to biology. Some of the world’s economically most disadvantaged countries harbor the majority of the planet’s biodiversity but are disproportionately understudied, and local research is often chronically underfunded^32–34^. Funding for both RADseq and AFLP studies appears biased towards economically rich countries, as shown in Fig. 1B. This bias is less severe in the case of the AFLP technique, which is often used in newly industrialized economies, such as India or Brazil (Fig. 1B). Institutions and scientists based in economically disadvantaged regions will be pivotal in dealing with the biodiversity crisis^35–37^. In this context, phylogeography, a discipline that is essential in defining conservation-relevant evolutionary entities and in addressing the taxonomic impediment, will be key^38–40^.

The perceived neglect to study “what is really out there”^41^ is by no means restricted to economically disadvantaged regions. A significant taxonomic bias is observable in both AFLP- and RADseq-based phylogeographic studies (Fig. 1C), and very species-rich groups, such as fungi, annelids, or echinoderms, are severely under-represented (Fig. 1C). In this context, it is especially worthwhile to explore the possibility that a well-established, cheap, and reliable tool such as AFLP could ultimately produce results that are as robust as genomic SNPs.

Here, we present a direct comparison between AFLP and RADseq by applying these two methods to the same dataset comprising six species of plants and arthropods co-distributed in the Eurasian steppes^42^. In all six taxa, a separation into at least two distinct groups reflecting the complex historic biogeography of Eurasian steppes has been revealed using RADseq in a large-scale study of Kirschner, Zaveska et al. 2020^42^. Specifically, we ask the following questions: (i) Do AFLP and RADseq retrieve similar phylogeographic patterns? Assuming that thousands of RADseq loci are more likely to resolve the phylogeographic structure of a species, we examine to what extent AFLP-based results reflect RADseq results. To evaluate possible differences between both methods, we applied phylogenetic network analysis and a set of similarity statistics. (ii) Does the information content in terms of phylogeographic information per locus of AFLP and RADseq results differ significantly? We evaluated the information content by resampling loci in a rarefaction approach for RADseq and by comparing them with AFLP markers. This allowed us to directly observe and compare the information content of the respective dataset. We will address both questions by applying our approach to six unrelated taxa with different evolutionary histories, genome sizes, ploidy levels, and spatiotemporal population dynamics. In addition, we produced a larger dataset for one of the six taxa for an in-depth analysis of the robustness of AFLP.

## Results

### Locus yield of AFLP and RADseq

After quality control, AFLP yielded between 100 and 600 fragments in the cross-taxonomic dataset containing all six taxa, and 985 for the large *Omocestus petraeus* dataset, containing only a single taxon (Table 1). The total number of RADseq loci in both datasets ranged from 5000 to 15,000 (Table 1).

**Table 1:** Locus yield before (in parentheses) and after quality control from amplified fragment length polymorphism (AFLP) for the six studied taxa and locus yield for restriction site associated DNA sequencing (RADseq) for the six studied taxa.

Method	N of loci		N of Individuals		Global R (ANOSIM)		R Mantel
	AFLP	RADseq	AFLP	RADseq	AFLP	RADseq	–
**Plants**							
*Astragalus onobrychis*	401 (1738)	10,606	18	15	0.903**	0.887**	0.773**
*Euphorbia seguieriana*	546 (1325)	13,335	18	16	0.826**	0.996**	0.636**
*Stipa capillata*	157 (1318)	10,959	17	17	0.175	0.92**	0.01
**Animals**							
*Omocestus petraeus* (cross taxonomic dataset)	486 (1680)	5946	15	15	0.567**	0.624**	0.479**
*Omocestus petraeus* (large dataset)	985 (1234)	7705	81	74	0.634**	0.634**	0.81**
*Plagiolepis taurica*	433 (1437)	6149	17	15	0.432**	0.758**	0.485**
*Stenobothrus nigromaculatus*	603 (1417)	5988	14	16	0.34*	0.588**	0.178

Global R values derived from Analysis of Similarity (ANOSIM)- and Mantel correlation coefficients calculated from the respective datasets and taxa; asterisks depict significance level (** = p > 0.01, * = p > 0.05).

### RADseq and AFLP contained similar information in four out of six studied taxa

**Figure 2:**
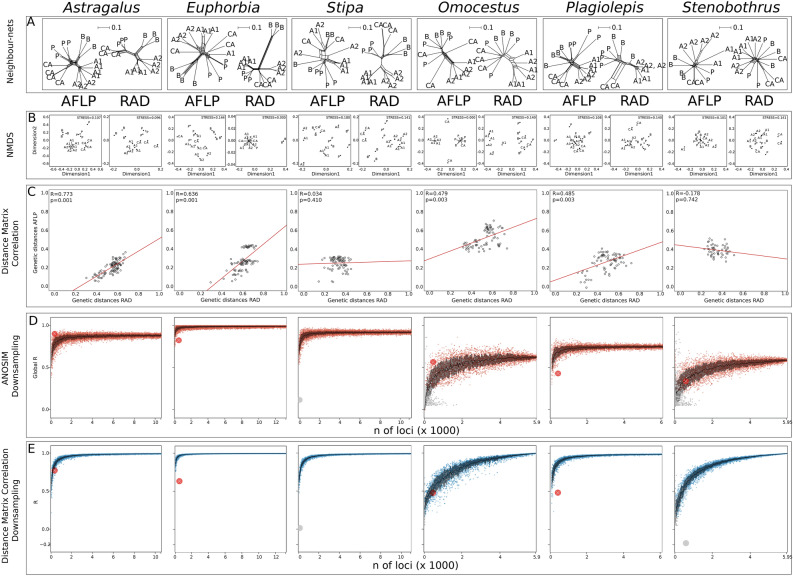
Comparative analyses for the six study species based on amplified fragment length polymorphism (AFLP) and restriction site-associated DNA (RADseq) sequencing data Abbreviations in A-B reflect the geographic origin of the sample: A1 = Western Alps, A2 = Eastern Alps, B = southeastern Balkan, CA = Central Asia, P = Pannonia. (**A**) NeighborNets inferred from AFLP data and RADseq data. (**B**) Ordination of AFLP and RADseq data via non-metric multidimensional scaling (NMDS). (**C**) Correlation plots of AFLP and RADseq derived distance matrices. Mantel R and corresponding significance levels are shown in the plot. (**D**) Global R values derived from analysis of similarity (ANOSIM)^79^ of down-sampled RADseq matrices. Each point represents one randomly down-sampled observation; colored points are significant observations, grey points are non-significant. Larger points show calculations based on the number of available AFLP loci. (**E**) Correlation coefficient R calculated via Mantel matrix correlation of down-sampled RADseq matrices and the full RADseq matrix. Each point represents one randomly down-sampled observation; coloured points are significant observations, grey points are non-significant. Large red points show calculations based on the number of available AFLP loci.

Information content of AFLP and RADseq markers was significantly correlated for *Astragalus onobrychis*, *Euphorbia seguieriana*, *Plagiolepis taurica* and *O*. *petraeus* (for both the cross-taxonomic dataset and the large *O*. *petraeus* dataset), as shown by the Mantel tests of intraspecific distance matrices derived from the respective datasets (Fig. 2C). In *Stipa capillata* and *Stenobothrus nigromaculatus*, however, this test showed no significant correlation (Fig. 2C). Therefore, comparative analyses concerning these two taxa were not interpreted any further.

**Figure 3:**
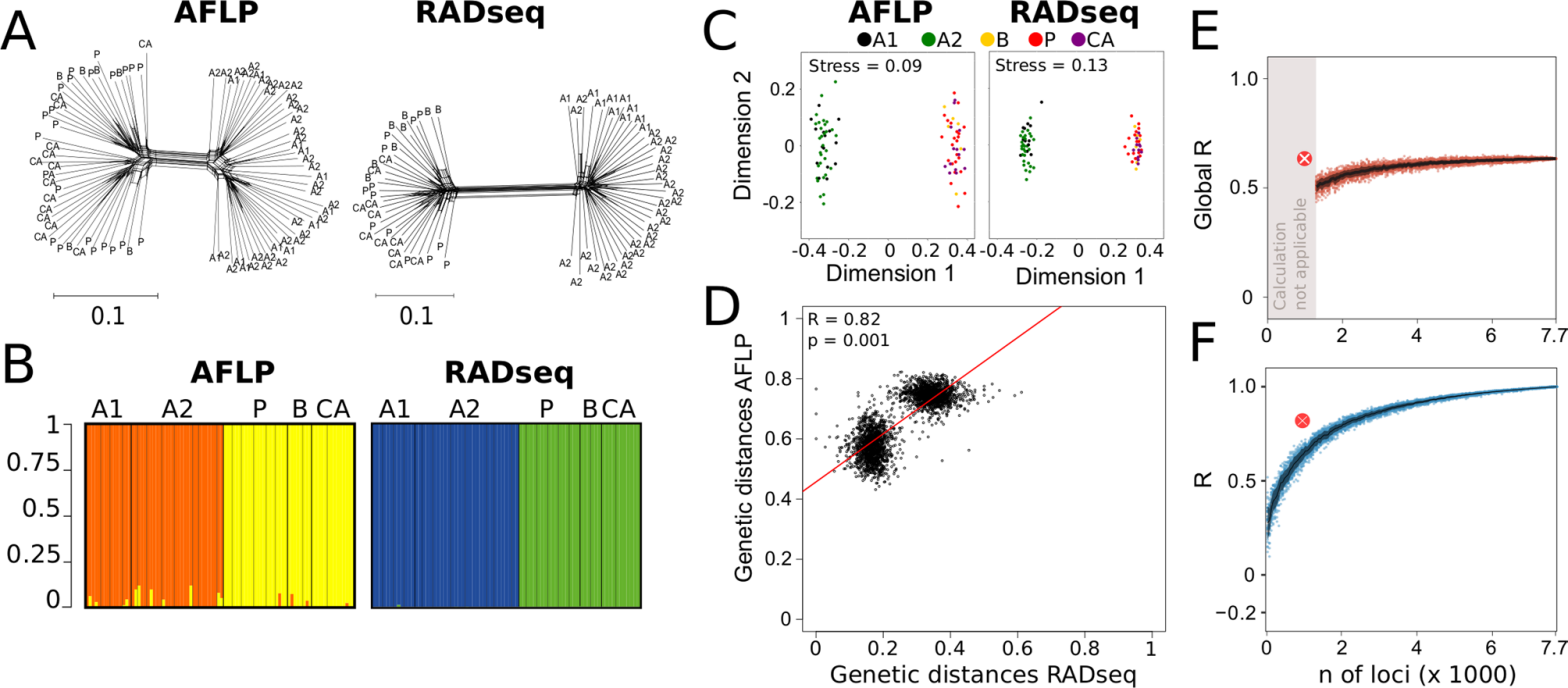
Comparative analyses based on the large *Omocestus petraeus* amplified fragment length polymorphism (AFLP) and restriction site associated DNA sequencing (RADseq) datasets. Abbreviations in A-C reflect the geographic origin of the samples: A1 = Western Alps, A2 = Eastern Alps, B = southeastern Balkan Peninsula, CA = Central Asia, P = Pannonia (**A**) NeighborNets inferred from AFLP data and RADseq data. (**B**) Bar plots show the two genetic groups identified via Bayesian clustering. Each bar represents one individual, colors show proportion of affiliation to a cluster. (**C**) Ordination plots of AFLP and RADseq data retrieved from non-metric multidimensional scaling (NMDS). (**D**) Correlation plots of AFLP and RADseq derived distance matrices. Mantel R and corresponding significance levels are shown in the plot. (**E**) Global R values calculated via analysis of similarity (ANOSIM) derived from the down-sampled RADseq matrices. Each point represents one randomly down-sampled observation; colored points are significant observations, grey points are non-significant. Large orange points show calculations based on the number of available AFLP loci. (**F**) Correlation coefficient R calculated via Mantel matrix correlation of the down-sampled RADseq matrices and the full RADseq matrix. Each point represents one randomly down-sampled observation; colored points are significant observations, grey points are non-significant. Larger points show calculations based on the number of available AFLP loci.

The presence of similar information content of AFLP and RADseq datasets was further illustrated by NeighborNet topologies and NMDS (non-metric multidimensional scaling) ordinations (Fig. 2A-B, Fig. 3A,C), which resulted for the cross-taxonomic dataset in similar backbone patterns with minor deviations (e.g. clustering of Central Asian samples in *O*. *petraeus*, Fig. 2A); or in the case of the large *O*. *petraeus* dataset, in the same clustering pattern (Fig. 3A). Analysis of similarity (ANOSIM) based on the geographic location resulted in similar between-region dissimilarities when using AFLP and RADseq data; an exception was *P. taurica,* where the AFLP-derived Global R value was much lower (Table 1).

The largest similarity in pattern and information content was observed between the large *O*. *petraeus* AFLP and RADseq datasets (Table 1, Fig. 3A,C,D). For these data, also Bayesian clustering analysis resolved two identical clusters irrespective of the data used (Fig. 3B, Supplementary Fig. 1).

### AFLP matched RADseq derived parameters under rarefaction in two out of six taxa

Random down-sampling of RADseq loci showed that ANOSIM-derived Global R values approached values inferred from the complete RADseq dataset when at least 2000 RADseq loci were subsampled from the original dataset (Fig. 2D). In some instances, even a lower number of loci led to similar results; however, smaller subsamples, led to large deviations (Fig. 2D). The largest deviations were observed in downsampling the small *O*. *petraeus* dataset. Here, Global R values were not fully converging, even with the full loci number as data basis (Fig. 2D). This effect was also observed in the down-sampled large *O*. *petraeus* dataset (Fig. 3E, Table 1). In the latter case, ANOSIM analysis was not even applicable when less than 1250 loci were subsampled due to the large quantity of missing data in the SNP matrix (Fig. 3E). Global R values calculated from AFLP data from *A*. *onobrychis* and *O*. *petraeus* (cross-taxonomic and large datasets) reached and even exceeded the Global R values of the corresponding downsampled RADseq dataset. However, in the case of *P*. *taurica* and *E*. *seguieriana*, the Global R values derived from AFLP did not reach the respective values obtained with the RADseq dataset.

For both plant species, Mantel correlation coefficients R estimated from the downsampled SNP matrices showed that full matrix similarity (R = 1) could be reached with approximately 1000 randomly-drawn RADseq loci (Fig. 2E). In the case of *P*. *taurica*, about 2000 loci were necessary to achieve this correlation coefficient, while for *O*. *petraeus*, matrix similarity was only gradually reached (cross-taxonomic & large datasets) (Fig. 2E). When AFLP distance matrices were tested for correlation with the corresponding downsampled RADseq based distance matrices, comparable and even higher matrix similarity coefficients were obtained in *A*. *onobrychis* and *O*. *petraeus* (Fig. 2D, Fig. 3F, Table 1). In *E*. *seguieriana* and *P*. *taurica*, the downsampled RADseq and AFLP Mantel correlation coefficients were smaller.

The amount of missing data in the full RADseq SNP matrices was randomly distributed after initial filtering and did not change in the performed random downsampling analyses.

## Discussion

We show that AFLP and RADseq-derived genomic data can yield similar phylogeographic patterns. Specifically, we employed a comparative phylogeographic framework to compare AFLP and RADseq datasets from six plant and animal species in terms of statistical similarity of phylogeographic patterns and information content. In four out of the six taxa, these two dataset-types were statistically similar in information content and resulted in nearly identical phylogeographic patterns (Figs. 2, 3). Surprisingly, a smaller dataset of AFLP loci (157 to 985) was akin to thousands of RADseq loci (5946 to 13,335) in their ability to resolve intraspecific genealogical patterns in these taxa. Compared with the RADseq-inferred results as benchmark, the robustness of AFLP based results in terms of information content and phylogeographic resolution is even more remarkable, especially when taking into account the distinct genome sizes (Supplementary Table 1), ploidy levels (diploid, tetraploid and octoploid populations in *A*. *onobrychis*^43^), evolutionary histories (taxa from two kingdoms and five different families) and spatiotemporal dynamics of the studied species.

In two species, however, AFLP loci failed to provide interpretable results. It might be that the locus yield (603 loci for the grasshopper *S*. *nigromaculatus* and 157 for the grass *S*. *capillata*) provided insufficient resolution, a point supported by the shallow phylogeographic structure detected with RADseq. We suspect that in the case of *S*. *nigromaculatus* the large size of the genome (11.36 giga base pairs) was responsible for the failure. An alternative explanation applicable to both species is the disproportionally high occurrence of repetitive elements in the genome, which has been reported for other grasses and Caelifera grasshoppers^44,45^. On the other hand, AFLPs have been able to resolve phylogeographic patterns in other studies of *S. capillata,* so a mere methodological issue in this study cannot be ruled out as error source^46^. However, this example highlights the limitations associated with the use of non-sequence-based genetic loci, such as the difficulties to infer the reasons behind method failure. It also highlights the importance of gathering information on biologically-dependent factors, such as genome size, especially when working with non-model organisms. If large genomes or a high proportion of repetitive elements in the genome are expected, scientists should refrain from using AFLP in favor of other techniques, such as RADseq. Because of these reasons, results from *S*. *capillata* and *S*. *nigromaculatus* are excluded from the discussion below.

Our results showed that AFLP performed equally well as RADseq when comparing the information content of dissimilarity matrices with regard to phylogeographic patterns (Global R) in two species (*A*. *onobrychis*, *O*. *petraeus*; Fig. 3D). Similarity of intraspecific dissimilarity matrices, however, did not increase when comparing downsampled RADseq datasets with AFLP (Fig. 2E). The downsampling revealed that in most cases fewer than 1000 RADseq loci were sufficient to reach the Global R of the full dataset. This indicates that in many cases a fraction of the SNP dataset might be sufficient to infer the phylogeographic structure within a species.

Generating an additional dataset for one species (*O*. *petraeus*), containing five times more individuals than the small cross-taxonomic dataset, enabled us to evaluate the influence of sample size on AFLP locus yield and, hence, the dataset information content. Compared with the cross-taxonomic dataset, the large *O*. *petraeus* dataset contained more AFLP loci (cross-taxonomic dataset: 486; large dataset: 985), and both dataset similarity and information content increased when compared with the corresponding large RADseq dataset (Fig. 3E–F, Table 1). As a consequence, the phylogenetic resolution of the large AFLP dataset was also better than in the cross-taxonomic AFLP dataset, and the incongruence observed in the latter (i.e. the clustering of some Central Asian individuals, Fig. 2A) disappeared (Fig. 3A). The downsampled AFLP dataset resulted in an even stronger support for the defined groups than the RADseq dataset (Global R, Fig. 3E).

A correlation between sample size (i.e. number of sampled populations per region) and phylogenetic resolution has been demonstrated in other RADseq-based phylogenetic studies^47,48^ and for several AFLP-inferred population genetic measures^49^. Small sample sizes and missing data might not be as much a problem for RADseq as for AFLP. Despite the large amount of missing data in the *O*. *petraeus* RADseq datasets, which hindered the calculation of Global R values from the downsampled data (Fig. 3E), the phylogeographic structure recovered by both RADseq datasets was similar, but this was not true for AFLP data (Figs. 2A, 3A). Given this, we emphasize here the need to include as many populations from the studied units (e.g. populations, regions) as feasible when using the AFLP technique. Comparing only a few populations is not only prone to produce ambiguous results but also limits data analyses. For instance, while we encountered convergence problems when analyzing the cross-taxonomic AFLP datasets with STRUCTURE^50^ (not shown), the same method worked well when applied to the large *O*. *petraeus* dataset and resulted in a similar clustering pattern, irrespective of the data type used (K = 2, Fig. 3B, Supplementary Fig. 1). However, the admixture observed in the AFLP-based clustering, and the lack of such signal in the RADseq based clustering, likely reflected noise in AFLP data rather than a true admixture signal. Similar to previous studies^49^, we found that small sample sizes can lead to ambiguous or erroneous results when inferring population structure from AFLP data.

While AFLP proved to be able to resolve intraspecific phylogenetic relationships in the study species, it is important to bear in mind that the phylogenetic methods utilized here are solely distance-based. In more complex scenarios, where large evolutionary distances between species and, consequently, large amounts of homoplasy are expected, distance-based phylogenetic methods in general, and the usage of AFLP in particular, are problematic^51^. In such scenarios, phylogenetic methods with underlying mechanistic models of molecular evolution, as implemented for example in most likelihood based approaches^52^, would be needed to adequately resolve phylogenetic relationships^53^. Such models of molecular evolution have been developed and extensively tested over decades of phylogenetic research and are easily applicable to DNA sequence data^53^ and, with some restrictions, to SNP data^26,54^, but not to AFLP. However, we want to point out that distance-based methods are solid options at the intraspecific or population level with low rates of overall change, which is the case in this study.

We emphasize that the intention of our study is not to advocate the use of allegedly old-fashioned alternatives to HTS techniques. On the contrary, we are convinced that the latter have revolutionized and will further revolutionize biology. It might be just a matter of time until these techniques will finally replace most “traditional” techniques that are still around, even if some of them, such as microsatellites, have shown to be remarkably resilient in competition against high-throughput sequencing techniques^55^. It is, however, unrealistic that the global scientific community is able to keep up in terms of methodology with economically rich labs working, for example, on questions in population and speciation genomics that require large sequencing depth and high-end bioinformatic analyses. While these players are perceived to constantly push the frontier, we should keep in mind that a substantial part of the theoretical basis of today’s population genomics originates from pre-sequencing days. Hence, the methodological toolbox available to a scientist can and should not be seen as a decisive factor to get papers published or projects funded, as long as the method applies to common standards, such as reproducibility. We think this is particularly important to bear in mind if we aim to extend the narrow taxonomic focus of current phylogeographic studies (Fig. 1C).

The methodological advancements around sequencing techniques are very dynamic, and methods praised until recently, such as RADseq, have also been criticized for their limitations, and might eventually be replaced by other techniques soon^56,57^. Given the pace of methodological developments that is obviously able to rapidly render methods obsolete, we highlight two points that we consider important. First, we showed that in a comparative phylogeographic scenario, meaningful phylogeographic patterns that were inferred via AFLP, an over 20-year old technique, survived a direct comparison with results inferred from RADseq. While the emphasized analytical and methodological limitations need to be considered, we show that AFLP data are robust and reliable. This is important concerning the backward compatibility of AFLP data in terms of significance of discoveries that have been made via this technique in the past. We emphasize that scientists that are successfully using this marker system in their lab, still have AFLP datasets, or simply cannot afford to switch to HTS methods right now should not be discouraged from using AFLP if the method is adequate to address the biological question asked. Secondly, many urging biological questions can be solved with simple methods. In other words, it might be unnecessary to obtain thousands of SNPs if a method like AFLP is sufficient to, for example, delimit phenotypically cryptic entities^58^, detect hybrid speciation^11^, or infer large scale phylogeography^12^. Facing the ongoing biodiversity and climate crisis, conservation policy-makers will need quick, large-scale, and straightforward answers^59^. To address this challenge, it will be crucial to strengthen the link of conservation genetics and conservation practice^60^. Thus, we want to emphasize that any source of robust molecular evidence should be seized in doing so.

In many scenarios, financial and personnel resource limitations are important arguments in favor of adapting the employed methodology to the specific question and not vice versa. As shown in this study, it is possible to robustly infer phylogeographic patterns by using AFLP. While advantages of high-throughput sequencing based methods like RADseq are obvious, we still want to encourage scientists and also publishers to maintain a critical stance towards the rampant method-centrism in an era of rapid methodological progress. Ultimately, the relevance of a result should be valued for its biological significance rather than the fanciness of the technique that was used to obtain it.

## Material and methods

### Taxon sampling and sample selection

**Figure 4:**
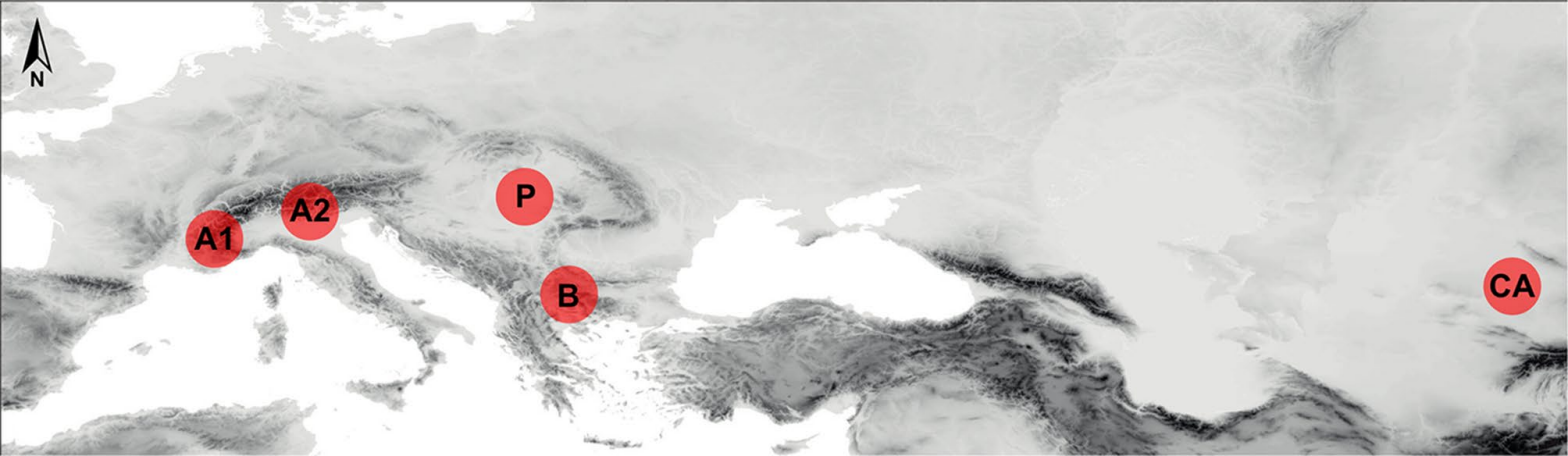
Map of Europe and Central Asia showing the sampling regions; A1 = Western Alps, A2 = Eastern Alps, B = southeastern Balkan Peninsula, CA = Central Asia, P = Pannonia. The underlying map is based on the GTOPO30 Global Digital Elevation Model (available at the United States Geological Service EROS Data Center, https://doi.org/10.5066/F7DF6PQS) and has been modified using QGIS version 3.10.5 (https://qgis.org/).

To cover a variety of evolutionary histories, three nominal species from three families of angiosperms, *Astragalus onobrychis* L. (Fabaceae), *Euphorbia seguieriana* Neck. (Euphorbiaceae), and *Stipa capillata* L. (Poaceae), and three arthropod species from two families, *Plagiolepis taurica* Santschi, 1920; (Formicidae), *Omocestus petraeus* (Brisout de Barneville, 1856), and *Stenobothrus nigromaculatus* (Herrich-Schäffer, 1840; both Acrididae), were selected. Samples of all taxa were collected between 2014 and 2016 from dry grassland localities from five regions, the Western Alps (A1), the Eastern Alps (A2), the Pannonian Basin (P), the southeastern Balkan Peninsula (B), and Central Asia (CA) (Fig. 4), in the course of a project to study the phylogeography of the Eurasian steppe biome^42^. Specimens were hand sampled and stored in silica gel (plants) or 96% ethanol (animals) for further analyses. For comparative analyses, distances among sampling localities for each taxon within the same region were always below 20 km, except the sampling localities of *S*. *nigromaculatus* in the Balkan Peninsula, which spanned 150 km. Three individuals per taxon and region were selected, resulting in a “cross-taxonomic dataset” of 90 samples for AFLP and RADseq analyses. In addition to this cross-taxonomic dataset, a second, single-taxon ”large dataset” was generated for *O. petraeus*. The latter comprised 81 individuals*,* evenly sampled from the five regions mentioned above.

### DNA extraction

Plant DNA was extracted from leaf tissue using a sorbitol/high-salt cetyltrimethylammonium bromide method^61^. The extract was purified using the NucleoSpin gDNA clean-up kit (Macherey–Nagel, Düren, Germany). Animal DNA was extracted from leg tissue (*O*. *petraeus* and *S*. *nigromaculatus*) or whole animals (*P*. *taurica*) with the DNeasy Blood & Tissue Kit (Qiagen, Düsseldorf, Germany). The same individual extracts were used for AFLP and RADseq experiments except for *Plagiolepis*, where extracts of two separate individuals from each nest had to be used due to the low DNA yield from a single individual.

### Amplified fragment length polymorphism fingerprinting

The AFLP protocol used for this study is described in detail in Wachter et. al.^62^. Briefly, DNA samples of all individuals were digested using the restriction enzymes *MseI* and *EcoRI*. Three randomly selected samples per species were added as "blind" samples to test for reproducibility and contamination, resulting in 18 replicated samples. For the large dataset, three replicates were randomly selected from each region, resulting in 15 replicated samples. Restriction digestion and ligation of the adapters was followed by pre-selective PCR amplification. The cycling conditions were: 2 min at 72 °C, followed by 30 cycles of 30 s at 94 °C, 30 s at 56 °C, and 2 min at 72 °C, and a final extension step of 10 min at 60°C^62^. For selective amplification, eight primer combinations (tEco-ACA/Mse-CAC; tEco-ACT/Mse-CTC; tEco-ACA/Mse-CAT; tEco-ATC/Mse-CTG; tEco-ACC/Mse-CAG; tEco-ACC/Mse-CAT; tEco-AAC/Mse-CAT; and tEco-AGC/Mse-CTG) were used, with each forward primer having a 5′ M13 tail (t). The cycling scheme was: 2 min at 94 °C, followed by 13 cycles of 30 s at 94 °C, 30 s at 65 °C decreased by 0.7 °C / cycle and 2 min at 72 °C, followed by 24 cycles 30 s at 94 °C, 30 s at 56 °C, and 2 min at 72 °C, completed by a final extension step of 10 min at 72°C58. FAM / HEX / NED / PET labelled M13 primers were added in the ratio M13:F:R = 10:1:10. Fragment analysis was performed by the Comprehensive Cancer Center DNA Sequencing & Genotyping Facility (University of Chicago, USA) on an ABI 3730 sequencer (Applied Biosystems, Chicago, USA).

### Scoring and quality assessment of AFLP markers

AFLP profiles were converted using Peakscanner v.1.0 (Applied Biosystems). Subsequently, optiFLP v.1.51^63^ was used in unsupervised mode to identify optimal parameters for scoring. The final peak scoring using the inferred parameters was done in tinyFLP v.1.40^64^. The three randomly selected replicates were used to assess whether a single AFLP locus carried correct biological information: in theory, the distance between these biological replicates should be zero (i.e., they should have identical AFLP profiles). In practice, a zero distance was rarely achieved, due to various factors introducing noise in peak scoring. When removal of a single locus from the binary AFLP matrix reduced the genetic distance between the replicated samples, this locus was considered to be affected by noise. In accordance with these considerations, a custom Python script (Supplementary Material) was used for the following procedure. First, the sum of p-distances between all pairs of replicated samples was determined. Then, the first locus was removed from the AFLP matrix, and the sum of distances was calculated again and compared with that of the full matrix. When the p-distance after locus removal was lower than in the full matrix, this locus was removed; otherwise the matrix remained unchanged. The same test was repeated for each locus. The script generated a new matrix file and a logfile with information on which loci were removed and how the sum of p-distances changed.

The R package vegan^65^ was used to calculate intraspecific distance matrices using Jaccard distances and visualize these matrices via non-metric multidimensional scaling (NMDS) adding 90% confidence interval ellipses. Based on these ordinations, individuals and/or primer combinations were excluded if an individual appeared outside the confidence interval in more than 50% of all primer combinations and if more than 10% of all individuals were outside the interval in a single primer combination.

### Restriction site associated DNA sequencing

Each taxon’s relative genome size was determined by flow cytometry^66^, whereby leaf tissue (plants), leg muscle tissue (*O. petraeus* and *S. nigromaculatus*), and whole heads (*P. taurica*) were used. Given the genome sizes, the desired sequencing depth and total fragment yield, the number of individually barcoded samples that could be pooled into a single RADseq library, and the optimal restriction enzyme for each taxon were assessed via RADseq counter^67^. RADseq libraries were prepared using a protocol modified from Paun et al.^68^. Per individual, 250 ng DNA (40 ng for *P*. *taurica*) was used for restriction digestion with the enzymes *Sbf*I (*O. petraeus* and *S. nigromaculatus*) and *Pst*I (plants and *P. taurica*). A double barcoding approach was chosen to decrease the number of adapters necessary to pool 96 individuals into a single library. A six-base-pair (bp) P2 barcode and an eight-bp P1 barcode, each differing by at least two bases from the respective barcodes belonging to the same adapter category, were selected to avoid erroneous assignment of fragments. P1 adapters (200 mM) were ligated to the restricted samples overnight at 16 °C. Samples were sheared in a two-minute-long, focused ultrasonication program using a sonicator (M220 series, Covaris Inc., Woburn, USA) to obtain average fragment lengths of 400 bp. To remove undesired fragment lengths from each pool, left- and right-side size selection steps were carried out, using × 0.7 and × 0.55 volume of SPRIselect reagent (Beckman Coulter, California, USA). After ligation of P2 adapters, samples were pooled. Before, the DNA content of each sample was quantified with a fluorometer (Fluoroskan Ascent, Thermo Scientific, Schwerte, Germany) using a fluorescent dye (Invitrogen Quant-iT PicoGreen dsDNA Assay Kit, Thermo Scientific, Schwerte, Germany). Samples were diluted to be equally represented in the final pool. Additional size selection steps were conducted on the left side of the target range using × 0.55 volume of SPRI reagent before and after the 18 cycles of PCR amplification with Phusion Master Mix (Thermo Fisher Scientific, Schwerte, Germany). The libraries were sequenced on a HiSeq2000 sequencer (Illumina, San Diego, United States) at the Vienna BioCenter (https://www.viennabiocenter.org/facilities/next-generation-sequencing/) as 100-bp single end reads.

### Identification of RADseq loci and SNP calling

Illumina raw reads were quality filtered and demultiplexed via the program process_radtags.pl, and RADseq tag catalogs were assembled and SNPs were called using the denovo_map.pl pipeline implemented in Stacks v. 1.46^69^. Large genomes, as observed in *O. petraeus* and *S. nigromaculatus*, are prone to contain large proportions of pseudo-genes, transposable elements and non-coding DNA^45^. To exclude such regions from the analyses, RepeatMasker^70^ was used to identify and mask repeated elements in the *Locusta migratoria* genome^45^ (GenBank: AVCP000000000.1), and the quality-filtered reads of *Omocestus* and *Stenobothrus* were then mapped to the masked *L*. *migratoria* genome using Stampy v1.0.20^71^. Only the raw reads that mapped on the masked *L*. *migratoria* genome were included in the final dataset.

Populations.pl^69^ was used to export SNP matrices in STRUCTURE^50^ format and phylip format^72^. Whitelists were used to exclude fragments with more than 10 SNPs, deleveraged stacks, and loci with more than 75% missing calls (85% in case of *O. petraeus*). To avoid violation of the assumptions of site-independent models developed for analysis of unlinked SNP data, we selected one random SNP per RADseq fragment using the write_random_snp flag for the Bayesian clustering analysis in STRUCTURE^50^. Phylip files were generated with the phylip_var option, which adds variant sites to the phylip output using IUPAC notation.

### Bayesian clustering analysis

Bayesian cluster analyses were done in the software STRUCTURE v.2.3.4.^50^. STRUCTURE runs for K = 1 to K = 5 were conducted, reflecting the number of sampled regions, with 10 replicates for each K using the default settings. Each MCMC ran for 2,000,000 generations, and the first 200,000 generations were discarded as burn-in. Bar plots and likelihood graphs were generated by the software CLUMPP v1.1.2^73^ and distruct v1.1^74^. The optimal number of clusters was determined following Evanno et al.^75^.

### Phylogenetic analyses and analyses based on intraspecific dissimilarity matrices

Phylip files based on RADseq data generated via populations.pl^69^, and 0/1 matrices based on AFLP data were used to calculate NeighborNets in SplitsTree4 v4.14.4^76^ using standard settings. NeighborNets were preferred over bifurcating neighbor joining trees, as networks provide a more complete picture concerning the pattern and the uncertainty of the retrieved splits.

The same matrices were used to calculate intraspecific dissimilarity matrices in the R package vegan^65^. To infer these distances within each dataset, Jaccard dissimilarities were used for AFLP data, and Gower distances for RADseq SNP data, as suggested for each data type^77,78^. For each species we were trying to assess (i) the similarity of AFLP and RADseq data and (ii) the role of geographic distance in the structuring of genetic variance among and within populations. To assess the first point (i) intraspecific distance matrices obtained from the AFLP and RADseq data were tested for correlation with Mantel tests, using the Pearson correlation method and 9999 random permutations in vegan^65^. To explore the second point (ii), the same R package was used to conduct Analysis of Similarity (ANOSIM)^79^, using geography (i.e. the sample region) as prior. Global R values returned by this analysis describe similarity within the defined populations and dissimilarity among them. Values close to 1 suggest similarity within populations and dissimilarity among them, while a value close to 0 indicates a lack of geographic structure among populations^79^. Finally, distance matrices were plotted in non-metric space via non-metric multidimensional scaling (NMDS). This was done in the R package vegan using the function that exhaustively iterates the scaling process until an optimal solution is reached^65^.

### Rarefaction

A random locus resampling approach was developed to assess the information content (i.e. how many loci are sufficient to obtain results similar to the full dataset) of RADseq data with decreasing locus number. Random resampling was done on modified SNP matrices exported via the structure flag in populations.pl^69^. Using custom R scripts, loci were sampled from the original SNP matrix in steps of 50 loci and 50 replicates per step, without sampling any locus twice in the same replicate. At each step, global R was inferred via ANOSIM, and a Mantel correlation test of the sub-sampled matrix with the full dataset was performed. The Global R and correlation coefficient R (Mantel test) obtained at each step were plotted using ggplot2^80^. Lines connecting the mean of each rarefaction step and the respective standard deviations were added to the plot. Global R and correlation coefficient R calculated from the corresponding AFLP dataset were added to the plots to compare the results with a correspondingly down-sampled RADseq dataset.

## Supplementary information

Supplementary Information 1.

Supplementary Information 2.

## Data Availability

RADseq data are available via the NCBI Sequence Read Archive (BioProject ID PRJNA680892). AFLP data are available in tabular format in Supplementary Data 1.
