# Supplementary material for: Performance comparison of two reduced-representation based genome-wide marker-discovery strategies in a multi-taxon phylogeographic framework
Source: Sci Rep. 2021 Feb 17;11:3978. doi: 10.1038/s41598-020-79778-x (PMC7889850; doi:10.1038/s41598-020-79778-x)
Supplement: Supplementary file 1 — Supplementary Information 1. [file 41598_2020_79778_MOESM1_ESM.docx]

# Performance comparison of two reduced-representation based genome wide marker discovery strategies in a multi-taxon phylogeographic framework

Kirschner Philipp, Arthofer Wolfgang, Pfeifenberger Stefanie, Záveská Eliška, Schönswetter Peter, The Steppe Consortium, Steiner Florian M., Schlick-Steiner Birgit C.

**Correspondence*: Philipp Kirschner, philipp.kirschner@gmail.com

# Supplementary Information

## Supplementary Methods

**Literature survey**

The number of publications using AFLP or RADseq was determined using the Web of Science Core Collection ([https://apps.webofknowledge.com](https://apps.webofknowledge.com/), accession date 09-04-2020), using the search terms “AFLP” and “amplified fragment length polymorphism”, and “RADseq”, “RAD sequencing“, “restriction site associated DNA sequencing” in the category “Topic” of the Basic Search mode. The results were downloaded in tabular format and sorted by publication year and funding agency. All publications were manually categorized according to the country hosting the funding agency. Countries hosting agencies that funded more than 10 projects were included in the final data set. Furthermore, title and abstract of all available RADseq publications were examined, and on this basis a selection that contained all studies done in a phylogeographic framework was made. This subset of studies was then grouped according to taxonomy of the study organism. A similar categorization was also done for the AFLP publications. For direct comparison, a similar categorization was also done for the AFLP publications. In detail, to achieve a sample size of AFLP studies equal to that of RADseq studies, publications were randomly drawn from the AFLP papers and included in the sample if they included studies done in a phylogeographic framework until the number of RADseq-based publications was reached.

Supplementary Results

**Literature survey**

The Web of Science query resulted in a total of 13,003 publications using AFLP and 1230 publications using RADseq. Categorization according to year of publication showed that AFLP usage reached its peak in 2011 and has gradually decreased since (Figure 1A). This decrease coincides with the increase of RADseq usage (Figure 1A); however, in 2019, AFLP was still more often used than RADseq. The categorization according to origin of funding agency showed that agencies from the US, the European Union, and China were the three top funding sources and provided money for 87% of all RADseq publications (Figure 1B). In the case of AFLP funding, the distribution was more even, while the same countries were the top three funding sources and provided funding for 71% of all publications (Figure 1B). A manual grouping resulted in 533 RADseq publications that were set in a phylogeographic framework. To retrieve an equal number of 533 AFLP publications with phylogeographic focus from the total AFLP publications, 2362 AFLP publications were randomly drawn and manually screened. Classification of these phylogeographic publications according to taxonomic group showed that the most frequently studied groups were chordates in the case of RADseq (49%) and vascular plants in the case of AFLP (71%) (Figure 1C).

Supplementary Table 1: Relative genome sizes of the taxa studied. The genome sizes are approximate values calculated relative to the genome size of reference species.

| Species | Relative genome size in giga base pairs |
| --- | --- |
| **Plants** |  |
| *Astragalus onobrychis* | 1.57 (diploid) 3.14 (tetraploid) 6.28 (octoploid) |
| *Euphorbia seguieriana* | 1.82 |
| *Stipa capillata* | 1.59 |
| **Animals** |  |
| *Omocestus petraeus* | 9.15 |
| *Plagiolepis taurica* | 0.52 |
| *Stenobothrus nigromaculatus* | 11.36 |


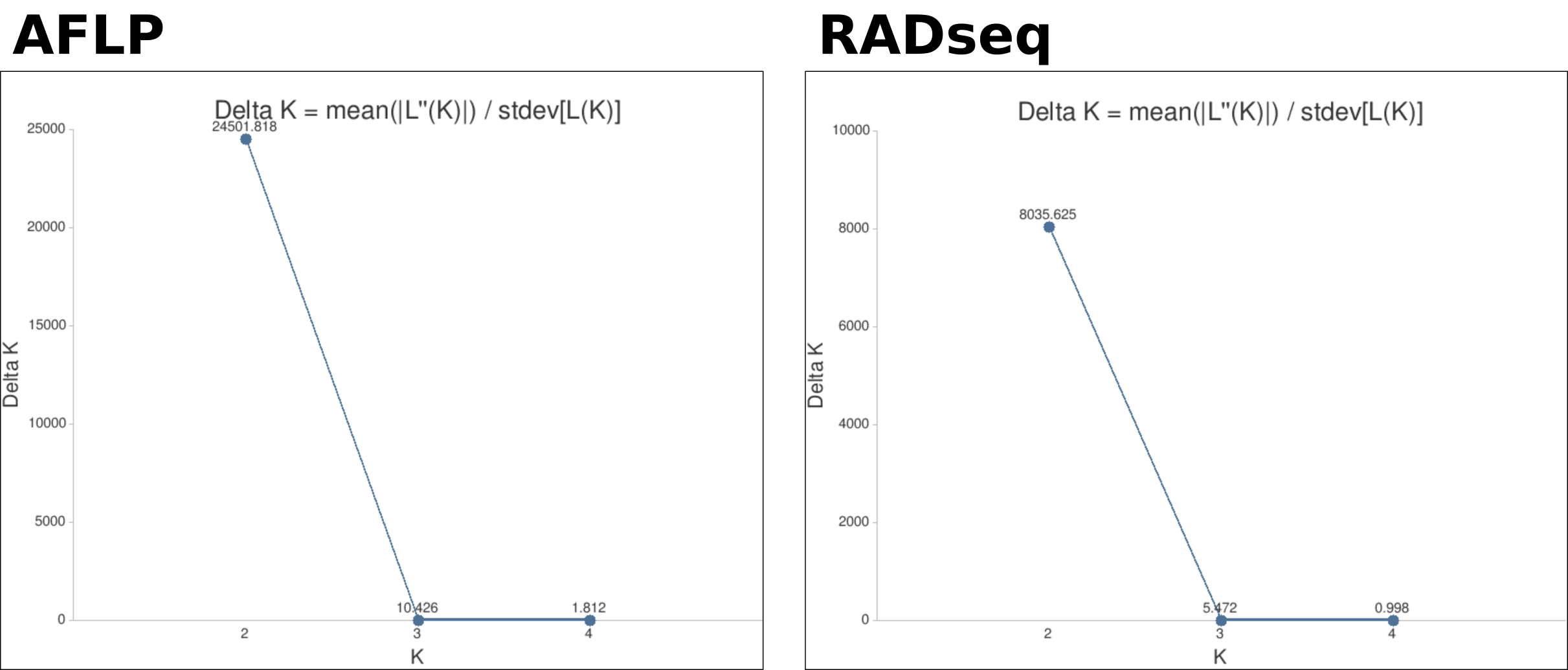


Supplementary Figure 1: Delta K values among runs of Bayesian clustering analyses based on the large *Omocestus petraeus* data sets, suggesting an optimal clustering into two clusters; amplified fragment length polymorphism (AFLP), restriction site associated DNA sequencing (RADseq) data sets.
